# The impact of a single round of community mass treatment with azithromycin on disease severity and ocular *Chlamydia trachomatis* load in treatment-naïve trachoma-endemic island communities in West Africa

**DOI:** 10.1186/s13071-017-2566-x

**Published:** 2017-12-28

**Authors:** Anna R. Last, Sarah E. Burr, Emma Harding-Esch, Eunice Cassama, Meno Nabicassa, Chrissy h. Roberts, David C. W. Mabey, Martin J. Holland, Robin L. Bailey

**Affiliations:** 1 Clinical Research Department, London School of Hygiene and Tropical Medicine, Keppel Street, London, WC1E 7HT UK; 2 Disease Control and Elimination Theme, Medical Research Council Unit The Gambia, P.OBox 273, Banjul, Atlantic Boulevard, Fajara, The, Gambia; 3 Programa Nacional de Saúde de Visão, Ministério de Saúde Publica, P.O. Box 50, Avenida de Unidade Africana, Bisssau, Guinea-Bissau

**Keywords:** *Chlamydia trachomatis*, Bacterial load, Spatial clustering, Trachoma, Disease severity, Community mass treatment

## Abstract

**Background:**

Trachoma, a neglected tropical disease, is caused by ocular infection with *Chlamydia trachomatis* (*Ct*). The World Health Organization (WHO) recommends three annual rounds of community mass drug treatment with azithromycin (MDA) if the prevalence of follicular trachoma in 1–9 year olds (TF_1–9_) exceeds 10% at district level to achieve an elimination target of district-level TF_1–9_ below 5% after. To evaluate this strategy in treatment-naïve trachoma-endemic island communities in Guinea Bissau, we conducted a cross-sectional population-based trachoma survey on four islands. The upper tarsal conjunctivae of each participant were clinically assessed for trachoma and conjunctival swabs were obtained (*n* = 1507). We used a droplet digital PCR assay to detect *Ct* infection and estimate bacterial load. We visited the same households during a second cross-sectional survey and repeated the ocular examination and obtained conjunctival swabs from these households one year after MDA (*n* = 1029).

**Results:**

Pre-MDA TF_1–9_ was 22.0% (136/618). Overall *Ct* infection prevalence (*Ct*I) was 18.6% (25.4% in 1–9 year olds). Post-MDA (estimated coverage 70%), TF_1–9_ and *Ct*I were significantly reduced (7.4% (29/394, *P* < 0.001) and 3.3% (34/1029, *P* < 0.001) (6.6% in 1–9 year olds, *P* < 0.001), respectively. Median ocular *Ct* load was reduced from 2038 to 384 copies/swab (*P* < 0.001). Following MDA cases of *Ct* infection were highly clustered (Moran’s I 0.27, *P* < 0.001), with fewer clusters of *Ct* infection overall, fewer clusters of cases with high load infections and less severe disease.

**Conclusions:**

Despite a significant reduction in the number of clusters of *Ct* infection, mean *Ct* load, disease severity and presence of clusters of cases of high load *Ct* infection suggesting the beginning of trachoma control in isolated island communities, following a single round of MDA we demonstrate that transmission is still ongoing. These detailed data are useful in understanding the epidemiology of ocular *Ct* infection in the context of MDA and the tools employed may have utility in determining trachoma elimination and surveillance activities in similar settings.

## Background

*Chlamydia trachomatis* is the leading infectious cause of blindness globally [1–3]. Trachoma is caused by infection with ocular strains of *C. trachomatis* and manifests as distinct clinical syndromes, beginning with an acute self-limiting kerato-conjunctivitis which may progress to chronic inflammatory disease with subsequent conjunctival scarring and blinding sequelae.

The World Health Organization (WHO) advocates the implementation of the SAFE strategy (Surgery for trichiasis, Antibiotics for active infection, Facial cleanliness to prevent disease transmission and Environmental improvement to increase access to water and sanitation) for trachoma elimination. Mass drug treatment with azithromycin (MDA) to entire communities aims to treat individual cases of infection and reduce the reservoir of infection, interrupting transmission within communities. Repeated episodes of conjunctival infection with *C. trachomatis* are thought to be required to cause the blinding sequelae of trachoma [2, 4]. Community-wide MDA, as part of the SAFE strategy, therefore aims to interrupt transmission, thus reducing the number of infections that each individual is exposed to and eliminate blinding trachoma as a public health concern [2, 5].

The WHO recommends between three and five annual rounds of MDA if the baseline prevalence of follicular trachoma in 1–9 year olds (TF_1–9_) at district level is between 10 and 39%, and at least five annual rounds of MDA if TF_1–9_ ≥ 40%, to achieve a reduction of TF_1–9_ to below 5% following treatment [5, 6]. These recommendations were made in 2010 after previous guidance [7] was found to be insufficient to eliminate trachoma. According to WHO guidance, trachoma control programmes should aim to administer antibiotics to at least 80% of the population. Following completion of MDA an impact survey is recommended, the results from the impact survey informing decisions about continuing treatment or conducting surveillance. There are conflicting data from trachoma-endemic communities on the optimal duration and mode of administration required to achieve the elimination target.

Oral azithromycin, even as a single dose delivered as a mass administration to communities, has significantly reduced the burden of active disease and in some populations has eliminated infection with *C. trachomatis* entirely [8]. However, the evidence-base relating to optimal frequency of MDA that will be effective in eliminating trachoma is not fully understood and may vary between settings [9].

In trachoma-endemic populations with an extremely high baseline prevalence of TF_1–9_, despite high coverage of MDA under research study conditions, levels of infection and disease, although reduced, can persist or return to pre-treatment levels. Longitudinal studies in Tanzania suggest that with present WHO protocols, hyperendemic countries may need yearly mass treatment for over ten years, which has significant economic and logistic impact on national trachoma programmes, non-government organisations and donors of azithromycin [10]. Similarly in Ethiopia, despite more than seven annual rounds of treatment in some regions showing that the prevalence of disease and infection can be reduced, but that on cessation of treatment, disease and infection return to baseline prevalence levels [11]. Despite adequate MDA coverage in line with WHO recommendations, disease elimination at 18 months post-MDA may not be sustained and re-emergence of disease within households has been demonstrated [12].

In some regions where annual treatment appears to have had little impact, even biannual treatment that has been implemented under research study conditions has failed to reduce TF prevalence in 1–9 year olds to less than 5% [13]. These data are supported by findings from a recent study, suggesting that extended MDA timelines may be required to interrupt transmission to meet elimination targets [14].

The dynamics of *C. trachomatis* transmission, particularly in the context of MDA, is complex and not well understood. Trachoma endemicity is likely to be important, as disease can disappear spontaneously from hypoendemic (TF_1–9_ 5–10%) communities [15–19] or disappear after a single round of MDA [8, 19]. In meso-endemic (TF_1–9_ 10–20%) populations disease prevalence may stabilize following MDA [12], and in hyper-endemic (TF_1–9_ > 20%) populations disease and infection persist despite multiple rounds of MDA [12].

The trachoma-endemic populations of the Bijagós Archipelago of Guinea-Bissau we have observed a strong association between *C. trachomatis* bacterial load and disease severity with spatial clustering of high load infections [20, 21]. In the current study we sought to assess the impact of a single round of MDA in these isolated treatment-naïve island communities on the prevalence of clinical trachoma and ocular *C. trachomatis* infection and investigate its effect on *C. trachomatis* load, disease severity and spatial clustering of *C. trachomatis* infection one year following MDA.

## Methods

### Study design and study population

The cross-sectional population-based trachoma survey methodology and this study population have been described previously [20–25]. Briefly, we used first stage cluster random sampling with geospatial representation on four islands at village-level to randomly select households for inclusion in the survey at baseline. A sample size of 1000 (including a design effect of 2 to account for anticipated household clustering) yields adequate power to estimate TF_1–9_ 5% ± 3% precision [26]. Two hundred and ninety-three households from all 38 villages were enrolled in the survey at baseline. Data were geo-coded at household and village level [20, 21]. One year following MDA we sought to follow up the households enrolled at baseline. A *de facto* census of each household was conducted at baseline and follow-up, from which individuals were enrolled.

### Clinical examination and conjunctival sampling

Clinical examination and conjunctival sampling were conducted at baseline and one year following treatment using standardised methods [20, 23]. A single validated physician examiner assessed each participant using the WHO simplified [27] and modified FPC [5] grading systems. In the modified FPC system, follicles (F), papillary hypertrophy (inflammation) (P) and conjunctival scarring (C) are each assigned a separate grade from 0 to 3. FPC grades of F2/3 or P3 equate to a diagnosis of active trachoma [TF (Trachomatous inflammation-Follicular) or TI (Trachomatous inflammation-Intense) by the WHO simplified system] and a grade of C2/3 (and in some cases C1) equates to a diagnosis of TS (Trachomatous Scarring). Clinical grading of the upper tarsal conjunctivae was conducted in the field as described previously [20–23]. The trachoma grader achieved an inter grader agreement (Cohen’s Kappa) score of > 0.9 against an international expert trachoma grader. Samples were taken using a polyester-tipped swab from the left upper tarsal conjunctiva of each participant using a well-tolerated standardized procedure [20–23]. Quality control swabs (pre-marked and drawn at random from the swab dispenser in the field) were passed 10 cm in front of the eye but without touching the eye were collected and treated in the same way as the conjunctival swabs for field and laboratory quality control. Our ddPCR assay enabled us to detect a human target (*Homo sapiens* RNase P/MRP 30-kDa subunit (RPP30) gene) to ensure that control swabs had not come into contact with the conjunctival surface and that swabs collected from participants were adequate samples (using strict minimum RPP30 detection criteria as described previously [22]). We found no evidence of cross-contamination using these methods; all control swabs were negative for *C. trachomatis* DNA and all conjunctival specimens were deemed adequate for inclusion in the analysis.

### Community mass treatment

A single height-based dose of oral azithromycin was offered by the national trachoma control programme distribution teams to all individuals in all communities participating in the study in accordance with WHO and national policy. Alternative treatment with tetracycline eye ointment was offered if there were contraindications to treatment with azithromycin. District-level coverage was estimated by the national trachoma control programme following the MDA using data from their MDA treatment registers and the most recent Electoral Census (2009).

### Detection and quantitation of *C. trachomatis*

DNA was extracted from swabs using QIAamp DNA Mini kit (Qiagen, Manchester, UK) and *C. trachomatis* DNA was detected and quantitated using droplet digital PCR (ddPCR) (Bio-Rad Laboratories, Hemel Hempstead, UK) as described previously [20, 22, 23]. Briefly, *C. trachomatis* plasmid-based ddPCR was used to detect DNA and diagnose infection and a single-copy pathogen chromosomal gene (*omcB*) was used to estimate pathogen load in each plasmid-positive sample [22, 23]. Estimated quantities of *omcB* (*C. trachomatis* load) are expressed as copies/swab.

### Statistical analysis

*Chlamydia trachomatis* quantitation data were processed as described previously [22, 23]. Data were double entered into a customised database (Microsoft Access 2007) and discrepancies resolved through source documents. Data were cleaned and analysed in STATA 13 (Stata Corporation, College Station, Texas USA). Statistical significance was determined at the 5% level.

We estimated the variance due to between-household, village and island clustering using null models for both active trachoma and *C. trachomatis* infection adjusted for age and gender in multivariable null models including all three cluster variables as previously described [22].

We examined trachoma and *C. trachomatis* infection prevalence data at baseline and follow-up using a Chi-square test of proportions. *Chlamydia trachomatis* load data were log_e_-transformed where indicated. Median load comparisons were made between baseline and follow-up using Kruskall-Wallis test. Associations between load and detailed clinical phenotype (defined by F and P scores using the modified FPC trachoma grading system) were examined using multivariable mixed effects linear and logistic regression models accounting for clustering detected in previous studies [20, 21] and adjusting for household *C. trachomatis* infection status at baseline in the follow-up analysis.

Geo-coded data were projected into UTM Zone 28 N and analysed in ArcGIS 10.1 (ESRI Inc., USA) [21]. A statistical measure of clustering (Moran’s I) was calculated at baseline and follow-up to evaluate the effect of MDA on the global spatial distribution of active trachoma and *C. trachomatis* infection. A local indicator of spatial association (Local (Anselin) Moran’s I) was used to identify clusters and outliers of *C. trachomatis* infection by load at baseline and follow-up. This method detects statistically significant clusters or outliers related to *C. trachomatis* load based on the calculation of *z*-scores for the distribution. Cluster types identified (relative to their *z*-score and *P*-value) include HH (high loads associated with other high loads), LL (low loads associated with other low loads), HL (a high load outlier associated with other predominantly lower loads) and LH (a low load outlier associated with other predominantly high loads). The geostatistical methods used are discussed fully elsewhere [21]. Briefly, we used the zone of indifference to define adjacency. This method assumes that each observation (individual *C. trachomatis* load) has local influence that decreases with distance beyond a critical distance cut-off, resulting in an adapted model of impedance, or distance decay, such that all features have an impact on all other features, but this impact decreases with distance. The crucial cut-off used in this study is derived from the distance over which spatial autocorrelation occurs in these data and relates to the village boundaries, assuming impedance as described above [21].

## Results

### Prevalence of trachoma and *C. trachomatis* infection

**Fig. 1:**
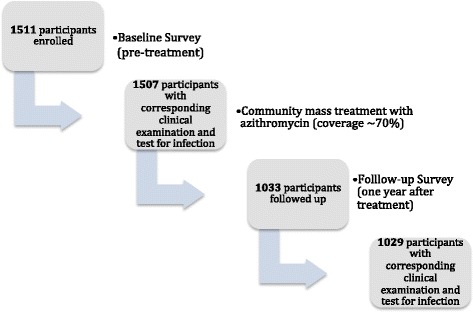
Enrolment of participants at baseline and follow-up one year after community mass treatment for trachoma control. Community mass treatment was distributed in accordance with WHO and national trachoma control policies. Zithromax® was donated by Pfizer Inc. through the International Trachoma Initiative

**Fig. 2:**
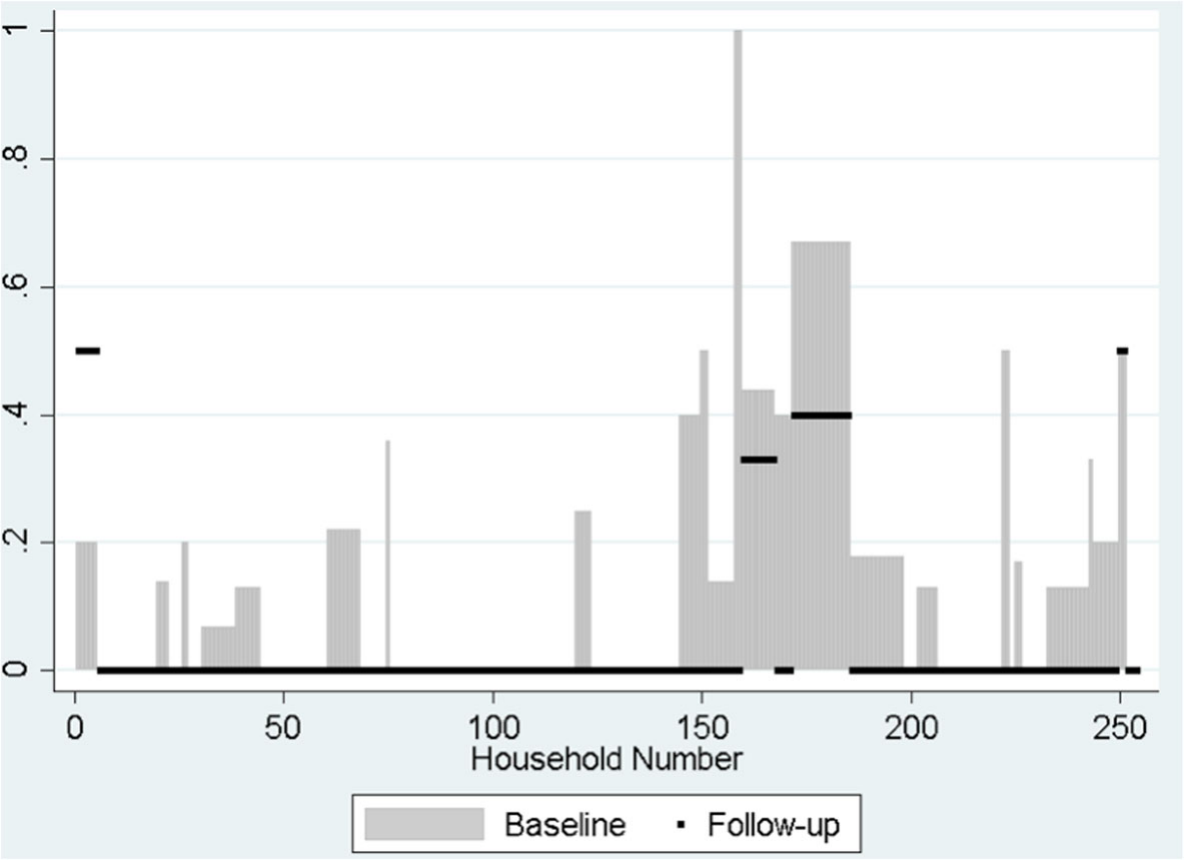
Prevalence of *C. trachomatis* infection in 1–9 year olds by household at baseline and follow-up. The grey bars show the prevalence of *C. trachomatis* infection (detected by ddPCR) in individual households at baseline. The black squares denote the prevalence of *C. trachomatis* infection in the same households at follow-up. The y-axis refers to the prevalence of *C. trachomatis* infection in 1–9 year olds. 13% (*n* = 37) households were not included at follow-up. Of those, almost half (*n* = 18) were households where *C. trachomatis* infection was detected in children aged 1–9 years at baseline

Participant enrolment and follow-up are illustrated in Fig. 1. MDA was conducted following the baseline survey and coverage was estimated at 70% (using district-level data provided by the national trachoma control programme) across the study islands. Socio-demographic characteristics did not significantly differ between individuals seen at baseline or at follow-up, nor were population-based estimates of the prevalence of scarring trachoma (TS) and trachomatous trichiasis (TT) (Table 1). The prevalence of TF and ocular *C. trachomatis* infection were statistically significantly reduced following treatment (Table 2). The prevalence of TF in 1–9 year olds was reduced from 22.0% (95% confidence interval, CI: 18.9–25.5%) to 7.4% (95% CI: 4.8–9.9%) (*P* < 0.001). The prevalence of TI in this age group was also reduced: from 2.9% (95% CI: 1.4–4.1%) to 1.5% (95% CI: 0.3–2.7%). The prevalence of ocular *C. trachomatis* infection was reduced in the population from 18.6% to 3.3% (*P* < 0.001) and in 1–9 year olds from 25.4% to 6.6% (*P* < 0.001). Figure 2 shows the household prevalence of *C. trachomatis* infection in 1–9 year olds in the households seen at both time points demonstrating that the follow-up prevalence is much reduced compared to baseline in the majority of households. However 13% (37/293) households were lost to follow-up, and of those almost half (18/37) were households where *C. trachomatis* infection was detected in 1–9 year olds at baseline.

**Table 1:** Study population characteristics at baseline and follow-up at one year

	Baseline	*n* ^b^	Follow-up	*n* ^b^
Median age (years) (IQR^a^)	13 (5–32)	1507	12 (5–35)	1033
Female	58%	869	63%	648
Age group				
0–5 years	28%	416	28%	288
6–10 years	16%	250	17%	180
11–15 years	11%	157	10%	113
> 15 years	45%	684	45%	452
Conjunctival scarring (TS^c^)				
Prevalence overall (95% CI)	23.8 (21.6–25.9)	357/1502	30.5 (27.7–33.3)	313/1026
Prevalence by age group (95% CI)				
0–5 years	2.7 (1.1–4.2)	11/414	2.1 (0.4–3.7)	6/288
6–10 years	2.8 (0.8–4.8)	7/250	5.6 (2.2–9.1)	10/177
11–15 years	11.5 (6.5–16.4)	18/157	18.9 (11.6–26.2)	21/111
> 15 years	47.2 (43.5–51.0)	321/680	61.3 (56.8–65.8)	276/450
Trachomatous trichiasis (TT_>15_^d^) (95% CI)	3.5 (2.1–4.9)	24/680	5.1 (3.1–7.1)	23/450

^a^IQR, interquartile range

^b^Denominator where indicated

^c^WHO Simplified Trachoma Grading System (28); TT_>15_ reflects TT in those over the age of 15 years

^d^CI, confidence interval

**Table 2:** The effect of community mass treatment with azithromycin on the prevalence of active trachoma and ocular *C. trachomatis* infection

Clinical category	Prevalence (%) (95% CI^a^)		Prevalence (%) (95% CI^a^)	
	Baseline	*n*	Follow-up	*n*
TF_1–9_^b^	22.0 (18.9–25.5)	136/618	7.4 (4.8–9.9)*	29/394
TI_1–9_	2.9 (1.4–4.1)	18/618	1.5 (0.3–2.7)	6/394
*Ct*_all_^c^	18.6 (16.7–28.8)	280/1502	3.3 (2.2–4.4)**	34/1029
*Ct*_1–9_	25.4 (22.0–28.8)	157/618	6.6 (4.1–9.0)***	26/395

^a^CI, confidence interval

^b^TF_1–9,_ TF (Trachoma-Follicular) in 1–9 year olds; TI_1–9_ = TI (Trachoma-Intense Inflammatory) in 1–9 year olds

^c^ *Ct*, *C. trachomatis* infection in the population overall and in 1–9 year olds 293 households were included at baseline and 254 at follow-up. Of the households lost to follow-up, 18 had *C. trachomatis* detected in children aged 1–9 years within the household at baseline. *C. trachomatis* infection status of the household at baseline was adjusted for in the follow-up analysis

*P < 0.0001; Chi-square test, *χ* ^2^ = 37.5, *df* = 1, difference 14.6% (95% CI: 10.2–18.8%)

**P < 0.0001; Chi-square test, *χ* ^2^ = 131.7, *df* = 1, difference 15.3% (95% CI: 13.0–17.6%)

***P < 0.0001; Chi-square test, *χ* ^2^ = 57.5, *df* = 1, difference 18.8% (95% CI: 14.3–23.1%))

### Clustering of active trachoma and *C. trachomatis* infection

There was evidence of increased clustering at follow-up of active trachoma and *C. trachomatis* infection at village-level and infection at household level (Table 3). The Moran’s I for C. trachomatis infection at baseline was 0.06 (*z* = 2.10, *P* = 0.0353) and 0.27 (*z* = 3.85, *P* = 0.0001) at follow-up indicating increased clustering following MDA.

**Table 3:** Clustering of active trachoma and *C. trachomatis* infection

	Active trachoma		*Ct* infection	
Cluster level	CE_Baseline_^a^ (95% CI SE)	CE_Follow-up_ 95% CI SE)	CE_Baseline_^a^ (95% CI SE)	CE_Follow-up_ (95% CI SE)
Household	1.08 (0.79–1.48)	0.89 (0.85–0.95)	1.35 (1.08–1.68)	1.52 (0.85–2.74)
Village	0.76 (0.50–1.14)	1.05 (0.56–1.96)	0.89 (0.65–1.21)	1.04 (0.56–1.96)
Island	0.47 (0.15–1.49)	0.34 (0.08–1.37)	0.42 (0.17–1.00)	0.19 (0.01–5.76)

^a^CE, cluster estimates obtained from age-adjusted mixed effects regression models for active trachoma and *Ct* (*C. trachomatis*) infection at baseline and follow-up. 95% CI (confidence intervals) of the SE (standard error) are quoted. Mixed effects models including all three cluster levels showed household to have the strongest effect. All cluster estimates were significant at the 1% level (Wald Chi^2^)

### *Chlamydia trachomatis* ocular load and disease severity

**Fig. 3:**
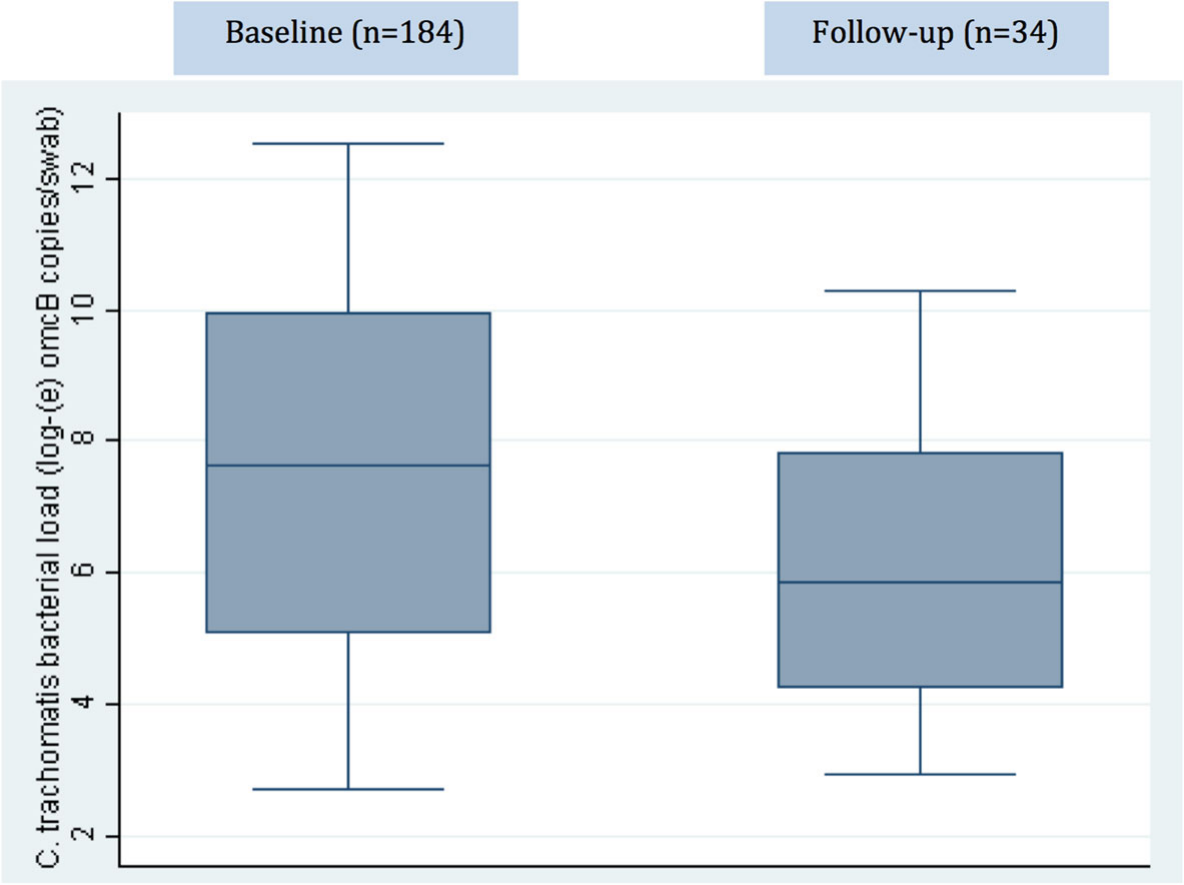
Reduction in median *C. trachomatis* load in ocular *C. trachomatis* infection following community mass treatment with azithromycin. Box-and-whisker plots showing the median *C. trachomatis* load (*omcB* copies/swab) from individuals with conjunctival infection at baseline and follow-up

**Fig. 4:**
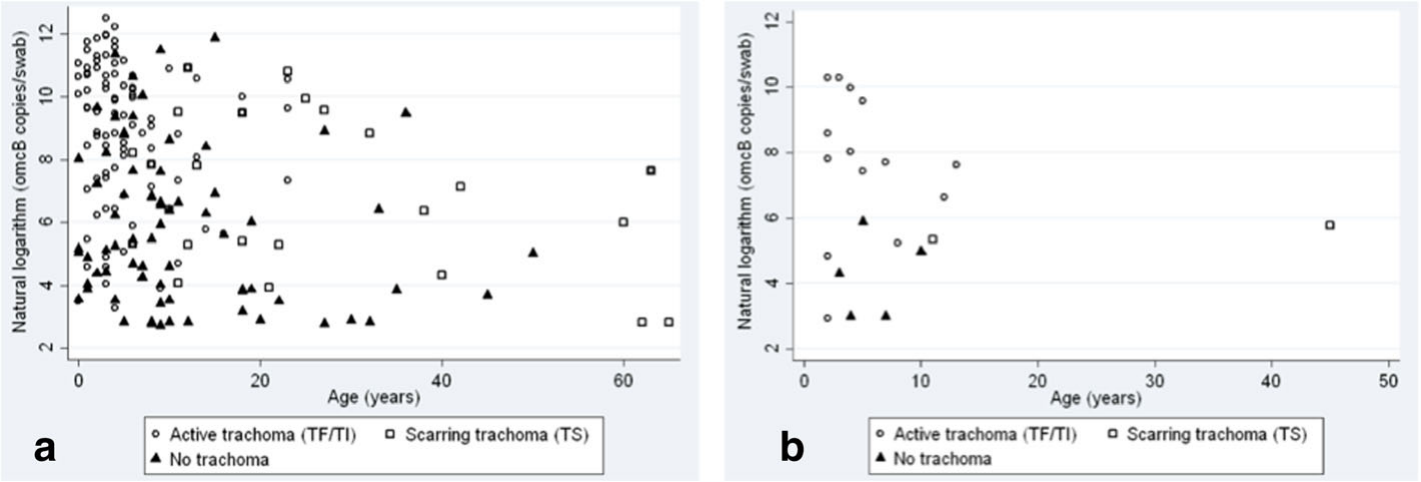
The effect of community mass treatment with azithromycin on ocular *C. trachomatis* load by age and clinical phenotype before treatment (**a**) and at one year following treatment with oral azithromycin (**b**). Clinical phenotype is defined using the WHO simplified grading system [27]

Median estimated load of *C. trachomatis* infection in infected individuals was significantly reduced from 2038 *omcB* copies/swab to 348 *omcB* copies/swab (*χ* ^2^ = 6.21, *P* = 0.0127) (Fig. 3). At follow-up almost all infections occurred in children under the age of 10 years, with 59% (20/34) occurring in children aged 0–5 years. At baseline a greater proportion of individuals with TS or normal conjunctivae were infected, indicating that infection was more widely spread in the population (Fig. 4).

**Fig. 5:**
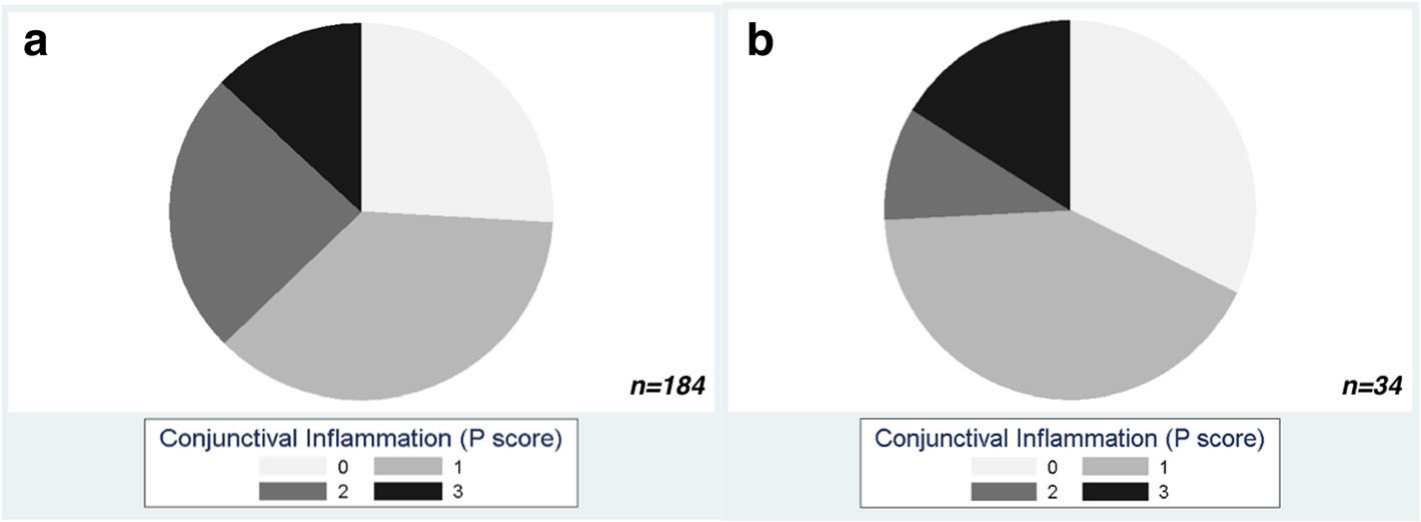
The effect of community mass treatment with azithromycin on conjunctival inflammation in individuals with ocular *C. trachomatis* infection. Proportion of individuals with conjunctival inflammation (P score 0–3 according to the modified FPC grading system at baseline (**a**) and follow-up (**b**)

In those with *C. trachomatis* infection, inflammatory disease was less severe at follow-up, most markedly with respect to conjunctival inflammation. There was a shift from higher proportions of P2 and P3 scoring disease at baseline to greater proportions of P0 and P1 scoring disease at follow-up (Fig. 5). However, age-adjusted mixed effect linear regression models accounting for household clustering and household *C. trachomatis* infection status at baseline demonstrate that a strong association remains between *C. trachomatis* load and conjunctival inflammation (P score), though the association is weaker at follow-up (OR_adj_ 11.65, 95% CI: 1.89–71.76) compared to baseline (OR_adj_ 27.6, 95% CI: 6.8–111.8) (Table 4).

**Table 4:** The effect of community mass treatment with azithromycin on associations between disease severity and *C. trachomatis* bacterial load in individuals with ocular *C. trachomatis* infection

		Baseline (*n* = 184)			Follow-up (*n* = 34)		
Clinical phenotype		*n*	OR_adj_ (95% CI)	*P*-value	*n*	OR_adj_ (95% CI)	*P*-value
F*-*score							
	F0	89	–		11	–	
	F1	22	1.67 (0.48–5.78)	0.417	5	2.15 (0.29–15.72)	0.450
	F2	27	1.84 (0.60–5.68)	0.287	6	3.56 (0.24–52.19)	0.354
	F3	42	6.16 (1.97–19.26)	0.002	9	17.71 (2.65–118.47)	0.003
P*-*score							
	P0	48	–		10	–	
	P1	68	6.15 (2.47–15.31)	< 0.001	13	1.25 (0.31–4.97)	0.753
	P2	45	21.74 (6.82–69.32)	< 0.001	3	1.16 (0.18–7.62)	0.875
	P3	24	27.61 (6.81–111.80)	< 0.001	5	11.65 (1.89–71.76)	0.008

*Note*: Age-adjusted multivariable mixed effects linear regression analysis of log_e_-transformed *C. trachomatis* load (*omcB* copies/swab) accounting for household clustering and *C. trachomatis* infection status at baseline (OR 0.98, 95% CI: 0.25–3.86, *P* = 0.975). F- and P-scores were assigned using the modified FPC grading system [5]. Individuals may appear in multiple clinical grading categories. *P*-value is for the Wald Chi^2^

### Spatial clustering of high load *C. trachomatis* infections

**Fig. 6:**
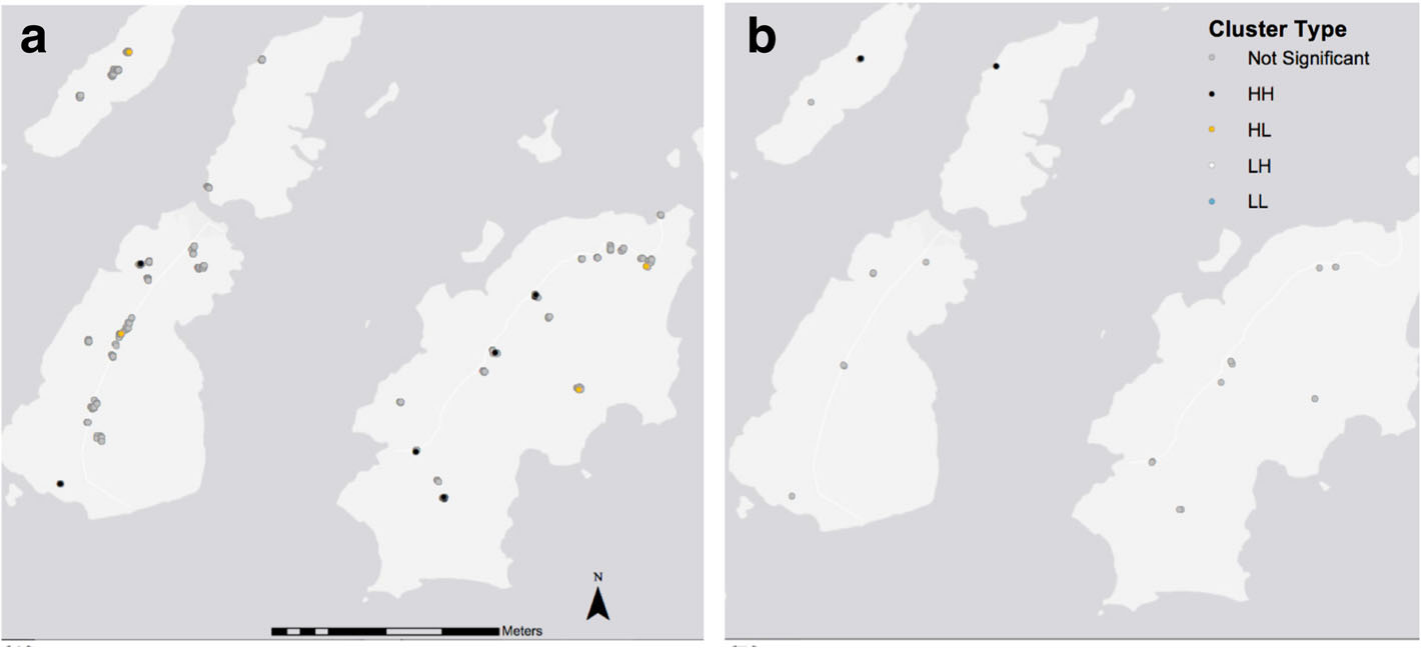
Cluster-Outlier maps showing the effect of community mass treatment on clusters of high load ocular *C. trachomatis* infections. *C. trachomatis* load was log transformed (ln(ln + 1)) due to significant negative skew. Statistically significant positive values for the Local Moran’s I statistic indicate clustering with similarly high (HH) or low (LL) values. Statistically significant negative values indicate that adjacent observations have dissimilar values and that this observation is an outlier (HL (a high load outlier) or LH (a low load outlier)). Maps are presented at baseline (**a**) and follow-up after MDA (**b**). HH clusters are observed at both time points. HL outliers are observed at baseline only. There are no LL clusters. Observation values represent *C. trachomatis* load

Maps were generated using the local Moran’s I statistic. These demonstrated clustering of *C. trachomatis* infection by load and found that at baseline there were a larger number of clusters of high load infections (HH clusters) than at follow-up. High load outliers (HL) were not present at follow-up. The HH clusters present at follow-up were at different locations compared to baseline. One HH cluster at follow-up was present in a location where there was an HL outlier prior to MDA. A second HH cluster was located where there was previously no clustering related to bacterial load. Clustering of low load (LL clusters) infections was not evident at either time point. Cluster-outlier maps at baseline and follow-up are presented in Fig. 6. The minimum value of *C. trachomatis* load observed within any HH cluster in this data set is ~10,000 *omcB* copies/swab irrespective of time point.

## Discussion

The WHO recommends that MDA aim for 80% coverage to be considered adequate for trachoma control programmes [5]. However, due to the significant logistic challenges that exist in this remote area it was only possible to deliver MDA to an estimated 70% of the population. Our data suggest that in these island communities, despite initial high disease and infection prevalence and suboptimal MDA coverage, we observed a dramatic reduction in prevalence of both TF in 1–9 year olds and ocular *C. trachomatis* infection (overall and in 1–9 year olds) one year after MDA. This type of dramatic reduction has been observed previously in treatment-naïve settings following azithromycin MDA [28].

Active trachoma and *C. trachomatis* infection (particularly those with the highest bacterial loads) were most prevalent in children under 10 years of age at both baseline and follow-up. Following MDA *C. trachomatis* infection virtually disappeared in adults and was reduced in those with scarred or normal conjunctivae. The presence of *C. trachomatis* infection across all age groups and clinical phenotypes at baseline is likely to represent the distribution typical of chronic endemic trachoma before MDA [29]. The reduction in the prevalence of infection and change in distribution by age and disease suggests a shift in the epidemiology of ocular *C. trachomatis* infection in these communities and may mark the beginning of control of transmission.

The significantly lower median ocular *C. trachomatis* load following MDA was consistent with findings from other studies suggesting that from 2 to 12 months following MDA the prevalence and load of infections remain low [12].

The number of clusters of high load infections detected using local spatial statistics was reduced and there was an absence of high load outlying infections amidst other low load infections after MDA. This phenomenon may be due to reduced chlamydial diversity in the population following MDA. The role of chlamydial strain diversity in transmission is unclear but greater diversity is likely to represent more successful transmission. There is some evidence that following MDA in other settings the number of *C. trachomatis* strains defined by *ompA* genotyping [30] or multi-locus sequence typing [31] was substantially reduced.

These data support the previous suggestion that *C. trachomatis* load is important in transmission of infection and its maintenance in the population [18, 21]. At both time points the minimum *C. trachomatis* load that we observed within an HH cluster was ~10,000 *omcB* copies/swab, supporting the hypothesis that there may be a threshold load important to sustain transmission, as suggested by Chidambaram et al. [32]. The change from an HL outlier prior to MDA to an HH cluster, and the appearance of a new HH cluster were there was previously none, following treatment suggests that there is likely to be ongoing transmission within this population. This particular location has a mobile population, being populated by fishermen and their families from Guinea Conakry, Sierra Leone and other islands on the archipelago. There are limited amenities in this settlement and it is possible that infection has been reintroduced. Introduction of infection following migration events has been documented in The Gambia [33]. In general, these island communities less susceptible to in-migration due to their isolated geographical location and therefore represent a unique opportunity to evaluate the effect of MDA in treatment-naïve trachoma-endemic populations.

Clustering of disease and infection was more apparent following MDA. Increased clustering of cases of *C. trachomatis* infection in treated communities has been described elsewhere [34]. In this study the strongest clustering of *C. trachomatis* infection was present at village level following MDA. This may be relevant in the context of previous spatial analyses conducted in this population suggesting that the village may an important unit of transmission in addition to the household in these communities and that the dynamics of transmission are different before and after MDA [21].

We used the detailed conjunctival grading system to investigate associations between infection, bacterial load and disease severity following MDA. A strong association between *C. trachomatis* load and inflammatory trachoma has been described previously [21, 28, 35]. The reduced association between infection and P score following MDA may reflect the decreased burden of circulating infection and decreased infection loads. It is likely that repeated episodes of infection are reduced following MDA due to a decrease in circulating *C. trachomatis* and subsequently reduced transmission. Moreover, in populations undergoing MDA there is evidence that clinical signs of trachoma become less specific for *C. trachomatis* infection [36, 37], suggesting that only the more severe phenotypes remain predictive of *C. trachomatis* infection. Azithromycin exhibits immunomodulatory effects that may be associated with reduced levels of inflammation [38], although at one year after a single dose this effect is unlikely to play a major role.

Although these are interesting data, the analysis is limited by the potential impact of loss to follow-up after MDA. 13% of households were not available at follow-up, and almost half of these were households where we found individuals with *C. trachomatis* infection at baseline. Clearly this may represent responder bias. The characteristics of the population in terms of age distribution, gender and chronic sequelae (trachomatous trichiasis) suggest that the follow-up sample is adequately representative of the baseline sample. However, the prevalence of conjunctival scarring in the cohort at follow-up was higher, possibly due to selective drop out of less severely affected individuals. This may affect the overall prevalence of *C. trachomatis* infection found at follow-up, as the prevalence of infection in conjunctival scarring is likely to be higher than in those with no clinical signs of trachoma [39]. Additional limitations of the study include the programmatic implementation of SAFE. We did not evaluate the implementation of the ‘F’ and ‘E’ components of SAFE to improve hygiene and sanitation alongside MDA in this study. Nor did we have access to accurate MDA coverage estimates, at individual or household level. At the time of the study there was limited implementation of ‘F’ and ‘E’ components of SAFE in Guinea Bissau, but there may still have been some effect in these communities that we could not evaluate [40]. Evaluating *C. trachomatis* load and clinical disease severity in cross-sectional studies is limited in the assumption that the duration of infection and the host conjunctival immune response are present in a steady state. To fully investigate the dynamics of *C. trachomatis* transmission more detailed longitudinal study is required, ideally in the context of individual or household level MDA coverage.

## Conclusions

In summary, through investigating the micro-epidemiology of *C. trachomatis* infection and its relationship with bacterial load and disease severity, these data suggest that MDA is likely to be having a significant impact on transmission of ocular *C. trachomatis* in these communities. However, further monitoring is required, as this geospatial analysis suggests that that there may be on-going transmission and risk of reintroduction of infection to communities despite MDA. The loss to follow-up in the population following MDA is also a concern and may indicate that these data underestimate the current burden of circulating ocular *C. trachomatis* infection and trachoma. Further longitudinal study, utilising mathematical models and high-resolution chlamydial genotyping and geospatial analysis, is necessary to provide a more complete picture of the relationship between disease severity, chlamydial load, transmission and elimination thresholds in communities undergoing MDA. These tools may improve our understanding of disease pathogenesis and transmission and may be useful in trachoma surveillance in post-MDA settings to identify clusters of infection and thresholds of *C. trachomatis* bacterial load that may be important foci of transmission.

## Abbreviations

*Ct*: *Chlamydia trachomatis*
ddPCR: Droplet digital PCR
FPC: Follicles, Papillary hypertrophy, Conjunctival scarring
MDA: Mass drug treatment with azithromycin
OR: Odds ratio
SAFE: Surgery for trichiasis, Antibiotics for active infection, Facial hygiene, Environmental improvements
TF: Trachomatous inflammation – Follicular
TF_1–9_: TF prevalence in 1–9 year olds
TI: Trachomatous inflammation – Intense
TS: Trachomatous Scarring
TT: Trachomatous Trichiasis
UTM: Universal Transverse Mercator coordinate system
WHO: World Health Organization
